# Classification Accuracy of Hepatitis C Virus Infection Outcome: Data Mining Approach

**DOI:** 10.2196/18766

**Published:** 2021-02-24

**Authors:** Mario Frias, Jose M Moyano, Antonio Rivero-Juarez, Jose M Luna, Ángela Camacho, Habib M Fardoun, Isabel Machuca, Mohamed Al-Twijri, Antonio Rivero, Sebastian Ventura

**Affiliations:** 1 Department of Clinical Virology and Zoonoses Maimonides Biomedical Research Institute of Córdoba Córdoba Spain; 2 Department of Computer Science and Numerical Analysis University of Córdoba Córdoba Spain; 3 Knowledge Discovery and Intelligent Systems in Biomedicine Laboratory Maimonides Biomedical Research Institute of Córdoba Córdoba Spain; 4 Faculty of Computing and Information Technology King Abdulaziz University Jeddah Saudi Arabia

**Keywords:** HIV/HCV, data mining, PART, ensemble, classification accuracy

## Abstract

**Background:**

The dataset from genes used to predict hepatitis C virus outcome was evaluated in a previous study using a conventional statistical methodology.

**Objective:**

The aim of this study was to reanalyze this same dataset using the data mining approach in order to find models that improve the classification accuracy of the genes studied.

**Methods:**

We built predictive models using different subsets of factors, selected according to their importance in predicting patient classification. We then evaluated each independent model and also a combination of them, leading to a better predictive model.

**Results:**

Our data mining approach identified genetic patterns that escaped detection using conventional statistics. More specifically, the partial decision trees and ensemble models increased the classification accuracy of hepatitis C virus outcome compared with conventional methods.

**Conclusions:**

Data mining can be used more extensively in biomedicine, facilitating knowledge building and management of human diseases.

## Introduction

Univariate and multivariate analysis are the two main conventional approaches to statistical analysis in the scientific method. Multivariate analysis in particular is used to determine the contribution of several factors (risk factors in biomedicine) to a single event or result. Genome-wide association studies (GWAS) have been widely used in case-control settings to identify which genetic variants, known as single nucleotide polymorphisms (SNPs), are associated with human diseases or traits [1,2]. In biomedicine, a number of studies have performed univariate and multivariate analyses based on the results of GWAS in order to obtain new risk or protective factors.

The 2017 study by our group using this method analyzed two groups of patients diagnosed with hepatitis C virus (HCV) infection [3]. One group consisted of patients who experienced spontaneous resolution of infection during first 6 months of infection (acute phase) and the other of patients who developed chronic hepatitis C. It is important from a clinical point of view to have tools available to predict HCV outcome (whether spontaneous resolution or chronic hepatitis C). With this in mind, one GWAS identified an SNP in the interferon lambda–3 (IFNL3) gene as a factor in spontaneous resolution [1]. That study showed that patients with the CC IFNL3 genotype had a greater likelihood of experiencing spontaneous resolution, while patients with the non-CC IFNL3 genotype were more likely to develop chronic hepatitis C. In our previous study, we studied whether haplotypes of the human leukocyte antigen (HLA) and killer cell immunoglobulin-like receptor (KIR) improved the predictive capacity of the IFNL3 genotype and found that different combinations of these genes (HLA-B44, HLA-C12, and KIR3DS1), together with the IFNL3 genotype, increased the classification accuracy of HCV outcome. More specifically, based on this combination of genes, a patient could be classified as having a genetically unfavorable profile (GUP) or a genetically favorable profile (GFP) for spontaneous resolution of HCV infection.

Data mining—the process of extracting hidden associations in datasets—is a promising trend in biomedicine and important for identifying factors that are never discovered by conventional statistical methods [4-6]. A number of studies have demonstrated the effectiveness of data mining techniques in biomedicine. Examples include the application of feature selection methods to reduce the dimensionality of biomedical problems [7], identification of key genes to improve the accuracy of classification models [8], and the use of big data techniques in scenarios where there is a large volume of data [9]. The aim of our study therefore was to reanalyze the dataset used in our 2017 study [3] using data mining approaches in order to find models that improved the classification accuracy of the genes studied.

## Methods

### Dataset Description and Data Preprocessing

This study was completed using a dataset from our earlier research [3], which was performed between 2013 and 2017 on 138 individuals, all of whom were HIV/HCV coinfected patients from the infectious diseases unit at the Hospital Reina Sofía in Cordoba (Spain). The patients were categorized as chronic hepatitis C or spontaneous resolution. Patients with spontaneous resolution were those who had undetectable HCV viral loads during the acute phase of infection and did not require specific treatment; patients with chronic hepatitis C were those who had detectable HCV viral loads after the acute phase and needed treatment to be cured. Further information about spontaneous resolution and chronic hepatitis C and an analysis of the IFNL3, KIR, HLA-B, and HLA-C genes is published elsewhere [3].

The dataset comprises 43 input features from different markers in every patient. The markers were IFNL3 genotype (1 feature), HLA-B (17 features), epitope Bw (1 feature), HLA-C (12 feature), and KIR genotype (12 features). The input features of each marker are shown in Table 1. To prevent great loss of information, the data from patients with missing values in any of the input features were completed using the k-nearest neighbors imputation method (k=3) before conducting the computational study [10]. This method finds the nearest neighbors to instances with missing values and fills in the gaps with the most frequent value in the nearest neighbors. A total of 46 of 138 patients included in this study had missing values in the features. The dataset is publicly available [11].

**Table 1:** Features of each variable.

IFNL3^a^	Epitope Bw^b^	HLA-B^c^	HLA-C	KIR^d^
CC^e^	Bw4	B*07	C*01	3DL1
Non-CC	Bw6	B*08	C*02	2DL1
—	Bw4/Bw6	B*14	C*03	2DL2
—	—	B*15	C*04	2DL3
—	—	B*18	C*05	2DL5
—	—	B*27	C*06	2DS1
—	—	B*35	C*07	2DS2
—	—	B*38	C*08	2DS3
—	—	B*39	C*12	2DS5
—	—	B*40	C*15	3DL2
—	—	B*44	C*16	2DP1
—	—	B*45	C*18	3DS1
—	—	B*49	—	—
—	—	B*50	—	—
—	—	B*51	—	—
—	—	B*52	—	—
—	—	B*57	—	—

^a^IFNL3: interferon lambda–3.

^b^Bw: Epitope Bw.

^c^HLA-B: human leukocyte antigen–B.

^d^KIR: killer cell immunoglobulin-like receptor.

^e^CC: Genotype CC.

### Determining the Best Subset of Features

To construct the models, we followed a procedure that has been effectively applied in recent studies [12] for selecting the subset of factors or input features that best describes the patient [13,14]. We first ranked the features in terms of relevance from highest to lowest, and then selected the best subset of features. The relevance of a feature was weighted according to its ability to distinguish the classes [15].

To avoid bias in the process of estimating feature relevance, 6 well-known feature estimation methods were used: gain ratio [16], information gain [17], symmetrical uncertainty [18], consistency [19], chi-squared [20], and relief-F [21]. These feature estimation algorithms are all supervised learning methods that use a priori classification to estimate the relevance of features but do not depend on the effectiveness of a classifier, so the biases of the learning algorithms do not influence the feature selection process. The filter methods evaluate the usefulness of a feature (or set of features) based on measures of distance, dependency, information, or correlation with data [15].

**Figure 1:**
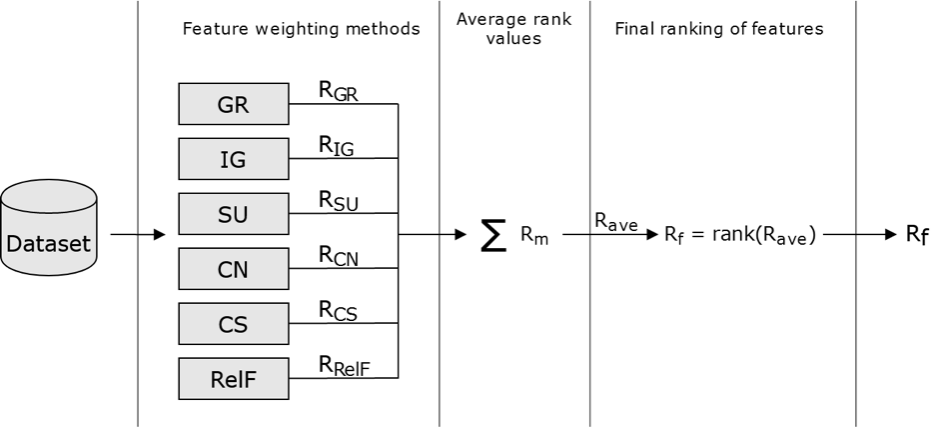
Flowchart illustrating the process of computing the final ranking of features.

To assess the feature weighting methods and better estimate the relevance of input features, the 10-fold cross-validation procedure was executed 3 times, and the results were averaged. The relief-F method was executed with parameter values set at between 5 and 10 [13,22]. The final ranking of features was computed as follows: (1) each feature weighting method provided its own ranking of methods, R_m_, with m being each individual method; (2) for each input feature, an average weight was computed as the average value of the rankings provided; (3) a final ranking, R_f_, was computed given the averaged weight values, in which the feature with the highest average weight was the most important. A flowchart of the process followed in this study to compute the final ranking is shown in Figure 1. This procedure was implemented using the open source WEKA (Waikato Environment for Knowledge Analysis) data mining software (v 3.9.3) [23].

Once the ranking of features was determined, it was then used to compute the subset of features that best predicted the class of a patient when constructing each model. We proposed a computational method that determined the best subset of features without evaluating all possible combinations of features. The combination formula used was 2^n^–1 (exponential size), with n being the number of features in the dataset. This method required evaluation of a maximum of 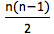 combinations of features.

**Figure 2:**
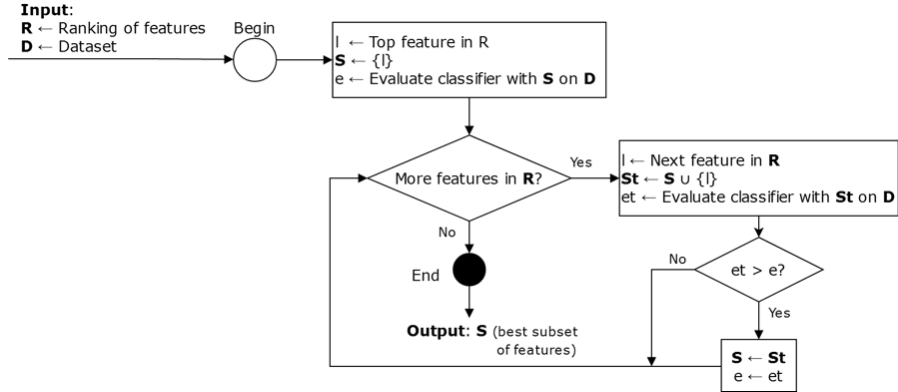
Flowchart to create classifiers with optimal subsets of features.

Given the final ranking of features R_f_, R_f_^l^ represented the subranking of features in which l was the highest ranked feature; in other words, the subranking of features was formed of l and all subsequent features in R_f_. Figure 2 shows a flowchart of steps performed for each R_f_^l^ subranking. Finally, each subset of features was evaluated according to the performance of the corresponding classifier in predicting whether or not a patient belonged to each group (chronic hepatitis C or spontaneous resolution).

### Model Construction

The procedure to select the best ranked subset of features was performed for each classifier used. We proposed the use of several classifiers, namely, partial decision trees (PART) [24], random forest [25], sparse linear discriminant analysis [26], support vector machines with both linear and Gaussian kernels [27], and L1-regularized logistic regression [28]. The aim of the classifiers is to learn from the input features and the outcome, for any new patient, is a prediction of whether the patient is categorized as chronic hepatitis C or spontaneous resolution. The random forest, sparse linear discriminant analysis, support vector machines, and L1-regularized logistic regression methods are regarded as noninterpretable (black-box) models; they usually perform better than white-box models and the outcome given by the model is always interpretable, even though it is not easy to understand the steps taken by the model to create its outcome or prediction. PART, on the other hand, is a white-box, rule-based classifier, which is more interesting from the point of view of interpretability of the model. PART returns a set of rules, one of which will be activated for each pattern. With respect to the set of rules, the expert can easily understand why it predicts each class; hence, although the performance of the final model is slightly reduced, it can provide the expert with valuable knowledge.

For the execution of each model, the 10-fold cross-validation procedure was repeated 3 times, evaluating on the corresponding test set in each case, thus averaging the values over a total of 30 different executions. For the algorithm parameter search, a random search of parameters was completed across 30 different combinations. The models were constructed using the caret package in R version 6.0-86 (R Foundation for Statistical Computing) [29].

### Selection of Best Models

To determine the effectiveness of the models constructed in the previous section, the area under the receiver operating characteristic (AUROC) curve was used. The AUROC of each model was obtained by averaging the AUROC on the corresponding test set over all 30 executions. All models with AUROC > 0.80 were stored for further analysis and study. In order to obtain a simple and interpretable model with high precision, we also highlighted and selected the PART model with the highest accuracy on the whole patient dataset for further study.

Ensemble learning is a widely used data mining task that enhances the final predictive performance of the classifier by combining the predictions of diverse simpler classifiers [30]. The use of ensemble models definitely reduces the interpretability of simpler models but usually yields much better classification performance. We proposed, therefore, not only to use the classifiers built so far but also a combination of some of them. To select members of the ensemble, we followed a similar procedure to the one used to obtain the best subsets of features. First, all single models were ordered according to classification accuracy over all patients. An ensemble was created with the first model only and then including the following model in the ensemble: if the resulting accuracy was better than before, the model remained in the ensemble, otherwise it was removed. Once all the models were tried in the ensemble, the procedure was repeated, but without using the best model. Finally, the subset of models that performed best together in the ensemble was returned.

### Comparison of Conventional and Data Mining Methodologies

Once the two models were obtained (the best PART model and the ensemble), their performance was compared with the results obtained from the previous study [3]. All the values that defined classification accuracy for chronic hepatitis C and spontaneous resolution in the models obtained in this study and the model obtained in the previous study (IFNL3, HLA-B*44, HLA-C*12, and KIR3DS1) were compared. These values included correct classification rate (CCR), positive predictive value (PPV) for spontaneous resolution, negative predictive value (NPV) for chronic hepatitis C, sensitivity, specificity, and AUROC.

The methods were compared in two analyses. The first analysis was based on all patients included in the study (n=138) and compared the classification accuracy of the IFNL3 genotype (CC or non-CC) against the models obtained in this study. A second comparison was then made that included only patients who had no missing values for the IFNL3, HLA-B, HLA-C, and KIR genes (n=104). The analysis consisted of comparing the classification accuracy of the models obtained in this study and combinations of genes obtained in the previous study. This combination of genes was used to classify a patient as having a GUP or GFP.

To avoid overfitting the training data for the models proposed in this paper, the data were split as follows. The dataset was partitioned using 10-fold cross-validation and the models trained using 9 out of 10 partitions, with the remaining one being used to evaluate the models. This was repeated so that all partitions were used once as test data. In this way, the models were evaluated using data from patients that had not been used during the learning phase, thus evaluating the ability of the models to generalize to new patients. In addition, the process was repeated on 3 different occasions using different seeds to create the partitions so that the results were consistent and not biased by the partitions created each time. Despite the lack of new data compared with the previous study, this process, which is standard in data mining, enables us to better determine whether the proposed models perform better than the previous one without this leading to overfitting the available data.

## Results

### Models Constructed for Spontaneous Resolution Prediction

After construction of the aforementioned classifiers from optimal subsets of features, we obtained more than 500 models with an AUROC greater than 0.80. A list of the models can be found in Multimedia Appendix 1. We focused first on the PART-1 model, which was constructed with just 4 different features: IFNL3, HLA-B*44, KIR2DS1, and KIR3DS1 (Multimedia Appendix 1). This yielded the best performance in terms of accuracy across all patients and was one of the simplest classifiers using the PART method, showing a good prediction performance but also being easy to interpret by experts. This method comprises a set of interpretable rules that are evaluated in order until one of them meets the conditions for a given example, as follows:

- IFNL3 = non-CC AND KIR3DS1 = no AND KIR2DS1 = no AND HLA.B44 = no: chronic hepatitis C (25.0/6.0)
- IFNL3 = non-CC AND KIR3DS1 = yes: chronic hepatitis C (20.0)
- HLA.B44 = yes AND KIR2DS1 = no AND IFNL3 = yes: chronic hepatitis C (12.0)
- HLA.B44 = no AND KIR3DS1 = no AND KIR2DS1 = yes AND IFNL3 = CC: spontaneous resolution (13.0/2.0)
- HLA.B44 = no AND KIR3DS1 = no AND IFNL3 = CC: spontaneous resolution (36.0/12.0)
- KIR2DS1 = no: chronic hepatitis C (11.0/2.0)
- KIR3DS1 = no AND HLA.B44 = no: spontaneous resolution (7.0/2.0)
- KIR3DS1 = yes AND HLA.B44 = no: spontaneous resolution (7.0/3.0)
- KIR3DS1 = no: spontaneous resolution (5.0)
- (default rule): chronic hepatitis C (2.0)

Given a patient, the decision algorithm first checks whether the conditions of the first rule antecedent are met; if they are, the patient is classified in the given class, otherwise, it tries the following rule. For example, the model first checks whether IFNL3 is “non-CC” and whether KIR3DS1, KIR2DS1, and HLA-B*44 are all “no.” If these conditions are met for the given patient, the patient is classified as chronic hepatitis C (because from the available data, 25 of the 31 patients who met these conditions were from the chronic hepatitis C class). If the patient does not meet any of these conditions, it checks whether the conditions for the second rule are met (if IFNL3 is “non-CC” and KIR3DS1 is “yes”), and so on. If none of the antecedents is satisfied, there is a default rule at the end for those patients who do not meet the conditions of any of the rules. Hence the model is highly interpretable by a clinician, since the model itself gives the reasons for its outcome.

Following the previously mentioned procedure, we also combined a subset of 5 models into an ensemble that included 3 random forest models (RF-25, RF-28, and RF-66) and 2 PART (PART-17 and PART-10). This combination of models was expected to perform better than the single PART model (and better than each of the members of the ensemble separately), although it was less interpretable than is the PART-1 method; in other words, it was more difficult to understand why this ensemble model returned each of its predictions. The ensemble model used a total of 24 features across all the base members: IFNL3, KIR2DS1, KIR2DS2, KIR2DL2, KIR3DL1, KIR3DL2, KIR3DS1, HLA-B*14, HLA-B*18, HLA-B*35, HLA-B*38, HLA-B*39, HLA-B*44, HLA-B*50, HLA-B*57, HLA-C*02, HLA-C*03, HLA-C*04, HLA-C*06, HLA-C*07, HLA-C*08, HLA-C*12, HLA-C*18, and Epitope Bw.

### Comparison of Accuracy Between the Methodologies

The classification accuracy for chronic hepatitis C and spontaneous resolution between the two methodologies (previous paper vs this study) was contrasted, and the results are set out below. Table 2 shows the results comparing the performance of the proposed methods and the IFNL3 marker using all 138 patients in the database. Table 3 compares the proposed models and the GUP/GFP model proposed in the previous study [3], in this case using only those patients without missing values for HLA-B*44, HLA-C*12, and KIR3DS1. Note that the proposed models are able to make predictions for these patients, but the GUP/GFP method is not, which is indeed one of the strengths of the models proposed in this study.

**Table 2:** Classification accuracy of IFNL3 (previous study) and models obtained (this study). Comparison made from the analysis of 138 patients without missing values for IFNL3.

Genotype/model	CCR^a^ %	PPV^b^ %	NPV^c^ %	Sensitivity %	Specificity %	AUROC^d^
IFNL3^e^ CC^f^/non-CC	71.0	61.6	81.5	78.9	65.4	0.72
PART^g^-1	78.6	69.6	87.3	84.3	74.8	0.85
Ensemble	82.5	77.7	86.8	81.2	83.7	0.89

^a^CCR: correct classification rate.

^b^PPV: positive predictive value for spontaneous resolution.

^c^NPV: negative predictive value for chronic hepatitis C.

^d^AUROC: area under the receiver operating characteristic curve.

^e^IFNL3: interferon lambda–3.

^f^CC: genotype CC

^g^PART: partial decision trees.

**Table 3:** Classification accuracy of genetically unfavorable profile/genetically favorable profile (previous study) and models obtained (this study). This analysis was performed on the 104 patients without missing values for any of the genes IFNL3, HLA-B, HLA-C, and KIR.

Profile/model	CCR^a^ %	PPV^b^ %	NPV^c^ %	Sensitivity %	Specificity %	AUROC^d^
GUP^e^/GFP^f^	76.0	64.4	84.7	76.3	75.7	0.76
PART^g^-1	80.0	69.6	87.3	84.3	74.7	0.85
Ensemble	84.8	77.7	86.8	81.2	83.7	0.89

^a^CCR: correct classification rate.

^b^PPV: positive predictive value for spontaneous resolution.

^c^NPV: negative predictive value for chronic hepatitis C.

^d^AUROC: area under the receiver operating characteristic curve.

^e^GUP: genetically unfavorable profile.

^f^GFP: genetically favorable profile.

^g^PART: partial decision trees.

In both cases, the proposed models using the data mining techniques outperformed the earlier study. The ensemble method returned a better performance than PART-1 on most evaluation metrics, although the virtue of PART-1 is that it is an interpretable model (see rules) and the clinician could obtain useful knowledge from the list of decision rules. In the case where all 138 patients were used, the ensemble raised the CCR in the model from 71.0% in the previous study to 82.5% and the AUROC from 0.72 to 0.89. In the comparison using 104 patients without missing values in their GUP features (see Table 3), the ensemble model likewise increased accuracy from 76.0% in the previous study to 84.8% and the AUROC from 0.76 to 0.89, thus consistently demonstrating a good level of performance relative to the standard approaches to this kind of data analysis.

Table 4 presents the results of all the metrics of each member in the ensemble method. The results demonstrate that combining the predictions of simpler but less accurate models leads to better overall performance.

**Table 4:** Classification accuracy of models included in the ensemble. Results obtained from the analysis of 138 patients without missing values for IFNL3.

Model	CCR^a^ %	PPV^b^ %	NPV^c^ %	Sensitivity %	Specificity %	AUROC^d^
rf^e^-25	77.1	74.9	79.5	69.4	83.5	0.88
rf-28	75.7	72.9	78.6	68.4	81.8	0.88
rf-66	76.0	70.2	81.2	73.7	78.8	0.85
PART^f^-17	78.6	74.1	84.6	78.7	79.1	0.79
PART-10	78.3	72.6	85.9	81.4	76.2	0.85

^a^CCR: correct classification rate.

^b^PPV: positive predictive value for spontaneous resolution.

^c^NPV: negative predictive value for chronic hepatitis C.

^d^AUROC: area under the receiver operating characteristic curve.

^e^rf: random forest.

^f^PART: partial decision trees.

## Discussion

### Principal Findings

In our previous study, we proposed a simple tool for the prediction of spontaneous resolution and chronic hepatitis C based on IFNL3 genotype and genetic profiles (GUP/GFP) using a combination of HLA-B, HLA-C, and KIR genes. In this study, a data mining methodology was followed to extract relevant hidden features, making it possible to identify subsets of relevant features that would provide greater precision when classifying patients into those who will go on to develop chronic hepatitis C and those who will experience spontaneous resolution of HCV infection. More specifically, in this study we present two models able to predict HCV outcome in each patient using just a subset of features: a simpler, interpretable one using just 4 features and a more complex one using 24 features. The study of both PART and ensemble models demonstrated that they yielded a much better predictive performance than the tool used in the previous study according to a number of different evaluation metrics such as CCR, PPV, NPV, sensitivity, specificity, and AUROC.

The factors analyzed in our previous study using conventional univariate and multivariate analysis showed that there was a strong association between IFNL3, HLA-B*44, KIR3DS1, and HLA-C*12 and the probability of developing chronic hepatitis C [3]. This study confirmed that the same factors were also important for HCV outcome (most of them are included in the PART-1 model and all of them in the ensemble model). In our previous study, however, we did not find any association with KIR2DS1, which is used in both models to predict which class the patient belongs to. This demonstrates that data mining is able to detect complex associations between factors, going beyond the analysis of individual factors commonly used in biomedicine. It is also interesting that the data mining methodology was able to identify genetic patterns hidden in univariate and multivariate analysis on the basis of a total of 138 patients. In the context of GWAS, many studies have found SNPs associated with pathologies without finding the mechanism or molecular basis to explain the associations, since the etiology of most human diseases is multifactorial and involves numerous genes. In this context, the data mining approach could facilitate the discovery of previously hidden genetic patterns in studies with high-dimensional data.

### Limitations

There are, however, certain differences in terms of the clinical applicability of the data mining approach depending on the type of model. In this study, for example, using PART, we were able to obtain an interpretable model (as in the previous study) and also a higher predictive performance. With the ensemble model, we obtained a higher predictive performance but lost interpretability (black-box model). Hence, if we are aiming to obtain the best possible predictive performance for patients, we should focus on the ensemble model. If the interpretability of the model is also of interest, since it gives the clinician useful information, the PART model would be preferred. More studies would be necessary to balance the accuracy of models against suitability for implementation in clinical decision making.

### Conclusion

Performance of data mining techniques in this study identified genetic patterns that were hidden by the conventional methodology using two models that increased the classification accuracy of HCV outcome. The data mining methodology could be used as an alternative approach in biomedicine, facilitating knowledge in the management of human diseases.

## Appendix

Multimedia Appendix 1 Area under the receiver operating characteristic of each model computed as the average value of 30 executions.

## Abbreviations

AUROC: area under the receiver operating characteristic
Bw: epitope Bw
CC: genotype CC
CCR: correct classification rate
GFP: genetically favorable profile
GUP: genetically unfavorable profile
GWAS: genome-wide association study
HCV: hepatitis C virus
HLA: human leukocyte antigen
IFNL3: interferon lambda–3
KIR: killer cell immunoglobulin-like receptor
NPV: negative predictive value for chronic hepatitis C
PART: partial decision trees
PPV: positive predictive value for spontaneous resolution
SNP: single nucleotide polymorphism
WEKA: Waikato Environment for Knowledge Analysis
